# Supplementary material for: A New Polycaprolactone-Based Biomembrane Functionalized with BMP-2 and Stem Cells Improves Maxillary Bone Regeneration
Source: Nanomaterials (Basel). 2020 Sep 8;10(9):1774. doi: 10.3390/nano10091774 (PMC7558050; doi:10.3390/nano10091774)
Supplement: Supplementary file 1 [file nanomaterials-10-01774-s001.pdf]

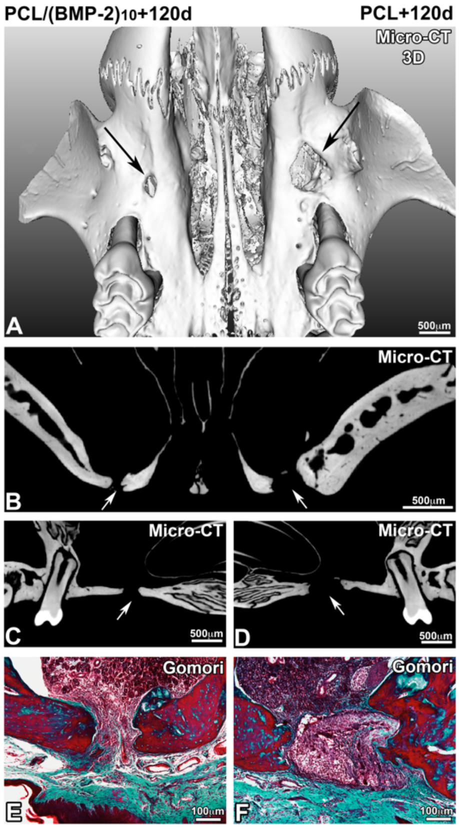

Supplementary Figure 1. Observation of bone regeneration of the maxillary bone after 120 days of implantation of a PCL membrane functionalized with BMP-2 (A, B, C, E) and non-functionalized PCL membrane (A, B, D, F). 3D reconstruction (A) of frontal (B) and sagittal (C, D) sections of X-ray microtomography and Gomori trichrome staining (E, F).
